# Modulation of the hepatic RANK-RANKL-OPG axis by combined C5 and CD14 inhibition in a long-term polytrauma model

**DOI:** 10.3389/fimmu.2024.1434274

**Published:** 2024-11-21

**Authors:** Yang Li, Klemens Horst, Johannes Greven, Ümit Mert, Ludmila Lupu, Annette Palmer, Lena Doerfer, Qun Zhao, Xing Zhang, Rebecca Halbgebauer, Anita Ignatius, Ingo Marzi, Martijn van Griensven, Elizabeth Balmayor, Frank Hildebrand, Tom Eirik Mollnes, Markus Huber-Lang

**Affiliations:** ^1^ Institute of Clinical and Experimental Trauma Immunology, Ulm University Medical Center, Ulm, Germany; ^2^ Department of Orthopaedics, Trauma and Reconstructive Surgery, Rheinisch-Westfälische Technische Hochschule (RWTH) Aachen University, Aachen, Germany; ^3^ Institute of Orthopaedic Research and Biomechanics, Ulm University Medical Center, Ulm, Germany; ^4^ Department of Trauma, Hand, and Reconstructive Surgery, University Hospital Frankfurt, Goethe University, Frankfurt/Main, Germany; ^5^ The Department of Cell Biology-Inspired Tissue Engineering (cBITE), MERLN Institute for Technology-Inspired Regenerative Medicine, Maastricht University, Maastricht, Netherlands; ^6^ Department of Immunology, Oslo University Hospital, University of Oslo, Oslo, Norway; ^7^ Research Laboratory, Nordland Hospital Bodø, Bodø, Norway

**Keywords:** polytrauma, RANK-RANKL-OPG pathway, immunomodulation, complement, CD14

## Abstract

**Background:**

Polytrauma and hemorrhagic shock can lead to direct and indirect liver damage involving intricate pathophysiologic mechanisms. While hepatic function has been frequently highlighted, there is minimal research on how the receptor activator of the NF-κB (RANK)/RANK ligand (RANKL)/osteoprotegerin (OPG) system is regulated in the liver following trauma. Furthermore, cross-talking complement and toll-like-receptor (TLR) systems can contribute to the posttraumatic response. Therefore, we investigated the hepatic consequences of polytrauma focusing on the RANK-RANKL-OPG axis, and evaluated the effects of a dual blockade of complement factor C5 and TLR-cofactor CD14 on hepatic features.

**Methods:**

The established pig model of polytrauma (PT) and hemorrhagic shock included pulmonary contusion, hepatic dissection, and bilateral femur fractures, surgically addressed either by external fixation (Fix ex) or intramedullary nailing (Nail). Four groups were investigated: 1) sham animals; 2) PT treated by Fix ex (Fix ex); 3) PT by Nail (Nail); or 4) PT by Nail plus combined C5/CD14 inhibition (Nail+Therapy). Serum samples were obtained between 0 - 72 h, and liver samples at 72 h after PT. Liver tissues were histologically scored and subjected to RT-qPCR-analyses, immunohistochemistry and ELISAs to evaluate the posttraumatic hepatic response with a focus on the RANK-RANKL-OPG system.

**Results:**

Following PT, the liver injury score of the Nail+Therapy group was significantly lower than in the Fix ex or Nail group without immunomodulation (p<0.05). Similarly, the degree of necrosis, lobular stasis, and inflammation were significantly reduced when treated with C5/CD14-inhibitors. Compared to the Nail group, AST serum concentrations were significantly decreased in the Nail+Therapy group after 72 h (p<0.05). PCR analyses indicated that RANK, RANKL, and OPG levels in the liver were increased after PT in the Nail group compared to lower levels in the Nail+Therapy group. Furthermore, liver tissue analyses revealed increased RANK protein levels and cellular immunostaining for RANK in the Nail group, both of which were significantly reduced in the case of C5/CD14-inhibition (p<0.05).

**Conclusion:**

Following experimental PT, dual inhibition of C5/CD14 resulted in altered, mainly reduced hepatic synthesis of proteins relevant to bone repair. However, a comprehensive investigation of the subsequent effects on the liver-bone axis are needed.

## 1 Introduction

Polytrauma is defined as an injury to two or more bodily parts due to violent factors, with at least one or the sum of all injuries being potentially life-threatening (1, 2). In the last decades two main surgical approaches have been established: damage control orthopedic surgery (DCO) with rather minimal invasive but only temporal surgery (e. g. by external bone fixation) and early total care (ETC) with definite but more invasive surgery (e.g. by intramedullary bone nailing), both of which are used in accordance to the overall trauma impact and pattern and patient’s condition (3). As treatment concepts continue to evolve and clinical practice progresses, trauma and its complications remain one of the leading causes of mortality, particularly among young and middle-aged individuals (4). The liver may not only bear the direct impact of trauma, but also - as the largest metabolic organ - become a central actor and target organ for the posttraumatic systemic inflammatory response (5). Various liver injury models with profound shifts in the hepatic transcriptome resulted in differential transcriptomic changes of extrahepatic organs, indicating a metabolite-mediated crosstalk between the liver and distant organs (6), including the bone. Regarding bone, both local and systemic inflammation are known to impair fracture healing (6–8). In our porcine polytrauma model, proteomic analysis of the fracture hematoma at 72 h post injury revealed the presence of coagulation-related, immunomodulatory, and osteogenic proteins, which were influenced by different surgical approaches. ETC using intramedullary nailing activated cellular and fluid components of while reducing the levels of proteins involved in osteogenesis and tissue remodelling. In contrast, DCO using external bone fixation led to elevated concentrations of proteins with anti-inflammatory and pro-regenerative properties within the fracture hematoma (9). Furthermore, severe concomitant trauma impairs fracture healing in mice (10) and men (11). Mechanistically, the initial temporal hypoxia seems to sustainably impact bone healing (12). However, the mechanisms of action of the proposed liver-bone axis after trauma still require further study.

Two crucial innate immunity recognition systems, namely the complement and the Toll-like receptor (TLR) systems, appear to play pivotal roles in trauma-induced inflammation (13). The complement system is vital for the hepatic homeostasis and immune response, influencing the development of various liver diseases, including alcoholic diseases and hepatocellular carcinoma, and hepatic ischemia-reperfusion injury (IRI) (14–17). Several complement inhibition strategies, including a C1- and a C3-inhibitor, as well as minocycline and doxycycline, have demonstrated effective attenuation of IRI to the liver following multiple injuries (18–20). CD14 is a co-receptor for several TLRs, in humans particularly for TLR4 and TLR2, and for mice it has been shown for several others (21). A number of studies on the TLRs and trauma has been published, but the CD14 molecule has barely been investigated, despite its important role as co-factor for several TLRs. One study showed that elevated soluble CD14 (sCD14) was a strong predictor for trauma patients who developed sepsis (22). Moreover, the combined inhibition of complement and CD14 as master alarm and processing systems of the systemic inflammatory response revealed significant anti-inflammatory effects (23).

Moreover, the combined inhibition of complement and toll-like receptor (TLR) as master alarm and processing systems of the systemic inflammatory response revealed significant anti-inflammatory effects (23). It is tempting to speculate, that such a combined blockade could eventually enable the surgeon to perform invasive ETC even when minimal-invasive DCO would be indicated.

However, the molecular mechanism of liver damage, induced either directly or indirectly after polytrauma (e.g. by combined pulmonary contusion, hepatic trauma and bilateral femoral shaft fractures or by the additional presence of a hemorrhagic shock) remain uncertain. In particular, the impact of various inflammatory and regenerative mediators released after liver injury requires further characterization, and especially further clarification to what extent they are dependent on the basic innate immune TLR- and complement system. In this context, it is also essential to elucidate the impact of traumatic liver injury and potential immunomodulation on pivotal factors that regulate bone fracture healing, including the receptor activator of NF-κB (RANK), its ligand (RANKL), and osteoprotegerin (OPG).

In the liver, RANKL and OPG are expressed by multiple cell types, including hepatocytes, Kupffer cells, and liver sinusoidal endothelial cells [3]. Furthermore, the RANK-RANKL-OPG axis represents a pivotal pathway in the context of liver injury and development of fibrosis (24). Additionally, hepatic stellatae cells (HSC) as drivers of fibrosis, also express RANKL, although its expression decreases as these cells activate (25). OPG, acting as a decoy receptor for RANKL, can inhibit the RANKL-RANK interaction, thereby suppressing the activation of HSCs and subsequent fibrotic response (26). The dynamic interplay between these components in the liver is complex and may link to the bone tissue (27) and can vary depending on the stage and type of liver disease. For instance, during the early stages of liver injury, RANKL may promote inflammation and fibrosis, while later stages might reveal a protective role through the inhibition of HSC activation (28). Understanding the RANK-RANKL-OPG axis may potentially offer novel approaches in managing liver injury and its complications, highlighting the importance of further research in this area (29).

Therefore, in the context of trauma, we specifically aimed to characterize the RANK-RANKL-OPG axis in the liver and systemically in a well-defined experimental polytrauma setting in absence or presence of an immunomodulatory therapy. We hypothesised that polytrauma results in an alteration of liver mediators relevant for inflammation and fracture repair; and, furthermore, that this response is improved by an immunomodulation approach targeting the central complement component 5 (C5) and TLR-coreceptor cluster of differentiation 14 (CD14).

## 2 Materials and methods

### 2.1 Animal model and group establishment

The study protocol of the hemodynamically instable pig polytrauma (PT) model was approved by the Office for Nature, Environment, and Consumer Protection of the State of North Rhine-Westphalia (LANUV AZ 81-02.04. 2020.A215) (17, 18). All pigs (German Landrace) from a pathogen-free barrier breeding facility, aged 12–16 weeks (weight 35 ± 5 kg), were maintained under a 12-h day/night rhythm for 7 d prior to the experiment to acclimatise to their surroundings.

A total of 25 animals were randomly distributed into four groups: sham (Sham, n=6) in absence of PT; simulated PT plus external fixation (Fix ex, n=8), PT plus internal fixation (Nail, n=7) and PT plus internal fixation plus combined C5/CD14 inhibition therapy (Nail+Therapy, n=4). The combined immunomodulatory therapy involved injecting C5 and CD14 inhibitors intravenously into the femoral vein. A C5 inhibitor (3 mg/kg body weight) was given 30 min after trauma to the animals in the therapy group, followed by a continuous infusion (1.1 mg/kg/h) until 72 h after trauma. The C5 inhibitor dose was based on titration and measured by effect on inhibitory complement activity in 3 pilot pigs observed for 8 hours (unpublished data). The pharmacodynamics of the C5 therapy in pigs in this study, allowing for accurate adjustment for 72 hours has been described in detail previously (30). The anti-CD14 inhibitor (mAb rMIL-2) was given at 5 mg/kg at 30 min, 12 h, and 30 h after trauma and at 2.5 mg/kg at 60 h after trauma. The dose was chosen based previous studies. In a porcine E. coli model, we titrated increasing doses to saturation of the CD14 molecules in blood leukocytes as measured by flow cytometry (14). The recombinant form of the original anti-CD14 (rMIL-2) used in this paper showed the same optimal dose of 5 mg/kg (31), and was used in success in a pig model of polymicrobial sepsis combined with C5 inhibition (32). This dose was therefore used in the present study.

All pigs were harvested 72 h after trauma and intensive care treatment. All data presented in the paper were obtained in the context of a larger study to address the 3R principles. Horst et al. (33) provided a specific description of the preparation and instrumentation. Azaperone (StresnilTM, Janssen, Germany; i.m. injection of 6-8 mg/kg body weight) and Ketamine (Ketanest, Pfizer, New York; 15 mg i.m./kg body weight) were used in combination as pre-anaesthetic agents. In addition, for general anaesthesia, Propofol (Fresenius, Bad Homburg vor der Hoehe, Germany; 2-12 mg/kg body weight/h) and Midazolam (Panpharma GmbH, Trittau, Germany; 0.02 -0.5 mg/kg body weight/h) were used for general anaesthesia and Fentanyl (Panpharma GmbH; 0.5-20 ug/kg body weight/h) was used as a general analgesic. Following intubation, PT was induced by a combination of injuries: blunt chest trauma, laparotomy with hepatic lacerations, haemorrhagic shock and bilateral open femoral shaft fractures. Systemic anaesthesia and analgesia as well as a lung-protective ventilation strategy with a tidal volume of 8–12 ml/kg body weight were applied throughout the experiment. Blunt chest trauma was induced on the right side of the pig’s chest during the inhalation phase using a bolt gun (Dynamit-Nobel, cartridge 9×17; Vienna, Austria) and a pair of steel and lead plates (0.8 and 1.0 cm thickness, respectively). Simulating clinical reality, the proportion of inhaled O_2_ was set at 21% for the first 90 min after trauma induction and was adjusted continuously thereafter according to real-time O_2_ saturation. Subsequently, a midline laparotomy was performed to expose the left liver lobe. Two incisions (4.5 cm × 4.5 cm) were made to the liver using a scalpel to simulate abdominal trauma. The bleeding was stopped after 30 s using a sterile gauze packing technique. Subsequently, the femoral shafts were exposed by a 5 cm skin incision. To induce bilateral femoral shaft fractures, a bolt gun and steel stamp were used at a 90° angle to the bone. Concurrently, pressure-controlled haemorrhagic shock was initiated by drawing blood from the femoral vein until a mean arterial pressure (MAP) of 40 ± 5 mmHg was obtained, or the maximum volume of blood drawn reached 45% of the total blood volume. This low MAP was maintained for 90 min. Throughout the 72 h of the experiment, the animals were given fluids (Sterofundin, B. Braun, Germany) at a rate of 0.5-2.0 ml/kg/h and parenteral nutrition (Aminoven, Fresenius Kabi, Germany) of 50-70 ml/kg body weight and day, under monitoring of the fluid balance. If required, norepinephrine was administered i.v. for maintaining the MAP>60 mmHg.

### 2.2 Drug introduction

UCB Pharma (Brussels, Belgium) provided RA 101295 (2-kDa peptide). This C5 inhibitor inhibits both C5 cleavage and the subsequent formation of the terminal complement complex C5b–9, which presents on the cell surface as the membrane attack complex (MAC).

RMil2 is a recombinant anti-pig CD14 antibody (clone MIL2; IgG2a), made available by Prof. TE Mollnes (Norway), that inhibits CD14-mediated pro-inflammatory cytokine responses. It is effective in porcine sepsis and IRI models when combined with a complement inhibitor (34).

### 2.3 Liver damage evaluation

Immediately after euthanasia, induced by deepening of the narcosis by 20 ml pentobarbital i.v. and a bolus of 40 ml KCl i.v., liver samples were removed and fixed in 10% formalin at room temperature (RT) for 24 h. Tissues were paraffin-embedded and sectioned onto slides. Ten images from random areas at 20× magnification were obtained from each slide. Morphological changes in haematoxylin and eosin (HE)-stained sections of the liver were examined by two independent blinded observers and scored accordingly. Stasis (appearance and distribution of red blood cells), vacuolation, parenchymal necrosis, and inflammation (appearance and distribution of polymorphonuclear granulocytes [PMNs]) were each graded into four features ranging each from 0 to maximal 3 points (0=none, 1=mild, 2=moderate, 3=severe). Thus, the overall histological liver damage score could range from 0 – maximal 12 points. Assessment of haemorrhage and necrosis was performed with a light microscope (Axio Imager M1, Carl Zeiss, Oberkochen, Germany) at a magnification of 2.5×. The number of liver tissue vacuoles and PMNs were assessed at 10× and 20× magnification.

### 2.4 Immunohistochemical staining of the liver

Formalin-fixed paraffin-embedded liver slides (4 μm thickness) were deparaffinized and rehydrated in a descending alcohol series. The antigen retrieval was performed in a microwave (700 W, 20 min) using citrate buffer (pH 6.0). The slides were blocked with 10% normal goat serum for 1 h at RT. Subsequently, the slides were incubated with a primary antibody (rabbit anti-pig myeloperoxidase (MPO) polyclonal antibody (Abcam) at a concentration of 1.4 μg/ml at 4°C overnight. MPO detection was based on an alkaline phosphatase method using a DAKO kit (Agilent Technologies). For RANK staining, the liver sections were similarly prepared and incubated overnight at 4°C with RANK primary antibody (Cloud-clone, Wuhan, China) at 1:100 dilution. Following washing with Tween 20 Tris-buffered saline, goat anti-rabbit horse-radish peroxidase-labelled secondary antibody (Abcam, UK) was added at 1:50 dilution at RT for 30 min and developed with DAKO (Agilent) for 20 min. Finally, the sections were visualised using a Zeiss Axio Imager A1 microscope. Seven images at 20× magnification were obtained from each slide and analysed using Image J automated cell-counting.

### 2.5 Real-time quantitative polymerase chain reaction

Liver tissue homogenates were centrifuged at 300 g for 5 min at 4°C and RNA was isolated according to the manufacturer’s (Qiagen Qiashredder™, Hilden, Germany) protocol. The RNA yield was quantified with a Qubit 2.0 fluorometer using the Qubit RNA BR Assay Kit (Thermo Fisher Scientific). cDNA was generated using the AffinityScript qPCR cDNA synthesis kit (Agilent Technologies) with oligo(dT) primers and stored at −80°C until further use. RT-qPCR was performed using a qPCR cycler Mx3000P (Agilent Technologies) and Brilliant III Ultra-Fast SYBR Green QPCR Master Mix (Agilent Technologies). Primers were purchased from Biomer.net (Ulm, Germany): glyceraldehyde 3-phosphate dehydrogenase (used as a housekeeping gene), OPG, RANK, and RANKL. Relative gene expression was determined by the 2^−ΔΔ^ Ct method (35) and results are reported as fold change compared to unstimulated control cells. Primers used for real-time PCRs are displayed in the **Supplementary Table**.

### 2.6 Enzyme linked immunosorbent assay

Serum and liver protein concentrations of RANK, RANKL, and OPG were determined using commercially available Porcine ELISA kits (RANK, RANKL: Mybiosource, San Diego, USA; OPG: Lsbio, Seattle, USA) using the pre-coated kits according to the manufacturers’ instructions.

For C5a measurements, a porcine ELISA (Hycult Biotech, Uden, Netherlands) was applied, strictly following the manufacturers’ protocol.

To determinate the total protein concentrations in serum samples and tissue homogenates, a commercially available bicinchoninic acid protein assay kit (Thermo Scientific, Rockford, USA) was used for protein determination as recommended by the manufacturer.

### 2.7 Aspartate aminotransferase measurement

Serum AST was analysed using an automated chemical analyser (VITROS 350; Ortho-Clinical Diagnostics, Raritan, NJ).

### 2.8 Data analysis

To check the normality of the obtained data sets, the Shapiro–Wilk and Kolmogorov-Smirnov tests were performed on each set of data. Unless otherwise noted, data satisfying normal distribution were expressed as the mean ± standard deviation (SD) or in the case of the histological analyses as the median with the 25^th^/75^th^ percentile. One-way Analysis of variance (ANOVA) followed by Holm-Sidak *post-hoc* testing was performed for comparisons between multiple groups. Data that did not satisfy a normal distribution were subjected to the rank sum test and *post-hoc* testing (Dunn’s method). A p<0.05 was considered to be statistically significant.

## 3 Results

### 3.1 Liver damage after experimental polytrauma is reduced by synchronic inhibition of complement C5 and CD14

**Figure 1:**
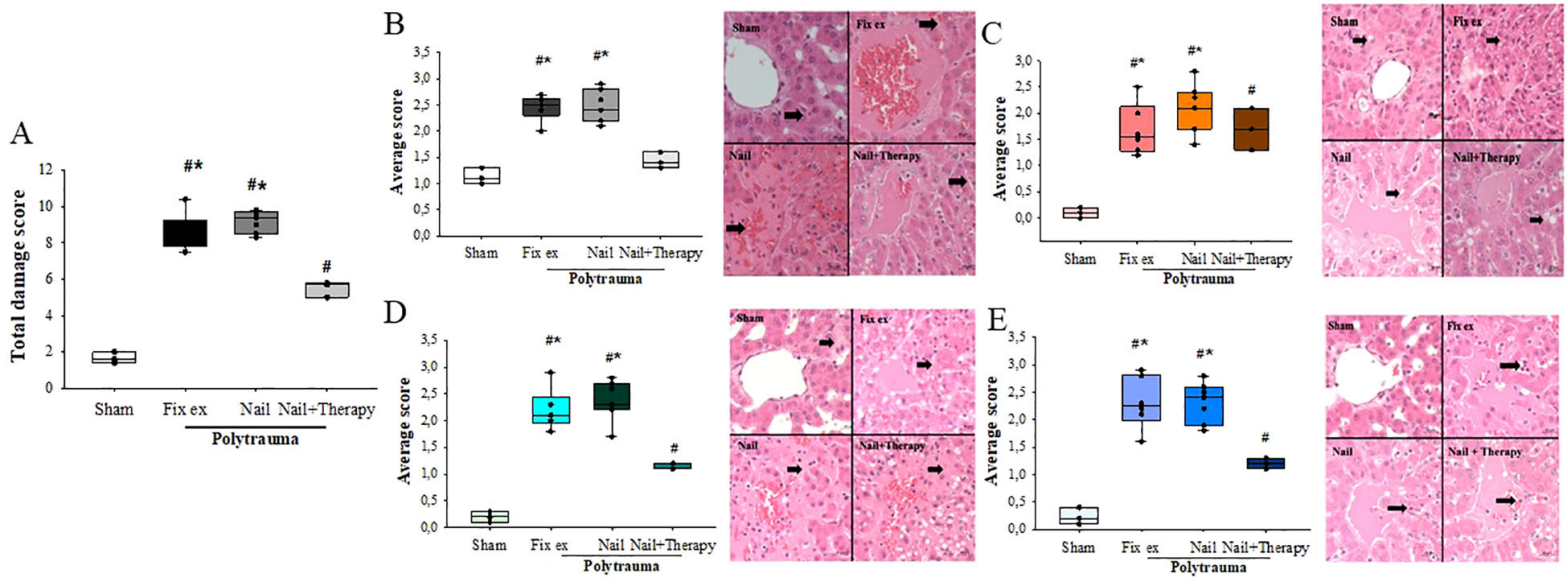
Polytrauma-caused increase in liver injury was improved by C5/CD14-immunomodulation. Liver histology (HE-staining, 400× magnification) was obtained from pigs in each group 72 h post polytrauma including hemorrhagic shock. Total liver damage score **(A)** for each group assessing four aspects: congestion **(B)**, vacuolation **(C)**, necrosis **(D)**, and inflammation **(E)**. The black arrow indicates the structure of the liver lobules. ANOVA with Holm-Sidak posthoc testing was applied to compare the data between the groups. Median values are displayed with the 25^th^ and 75^th^ percentile, respectively; denotes significant differences vs. Sham group, * denotes significant differences vs. Nail+Therapy group. Fix ex = external fixation, Nail = internal fixation with intramedullary nail, Nail+Therapy = intramedullary nail with combined C5/CD14 inhibition. Sham: n = 6, Fix ex: n = 8, Nail: n = 7, Nail+Therapy: n = 4.

Analysis of the HE-stained liver tissue sections taken 72 h after the polytrauma impact revealed a significant increase of the damage score in both the Fix ex and Nail groups versus the Sham group. The total score was 1.67 ± 0.31 for the Sham group, 8.65 ± 1.01 for the Fix ex group, and 9.19 ± 0.60 for the Nail group. By contrast, the liver injury score of the Nail+Therapy group (5.50 ± 0.44) was significantly lower than in the Nail or Fix ex group without the immunomodulatory therapy (**Figure 1A**). HE staining of the liver tissue in the PT groups revealed increased signs of disorganisation and congestion of the hepatic lobular structure (**Figure 1B**) and numbers of vacuoles (**Figure 1C**) and necrosis of some hepatocytes (**Figure 1D**) compared to the normal control group. An infiltration of neutrophils in the liver indicated a clear inflammatory response in the liver (**Figure 1E**). By contrast, the degree of necrosis, hepatic lobular stasis and neutrophil recruitment were significantly reduced in the Nail+Therapy group, which was not the case in the Nail or Fix ex groups without immunomodulation.

### 3.2 Effects of C5/CD14-inhibition on enhanced liver enzyme concentrations after polytrauma

**Figure 2:**
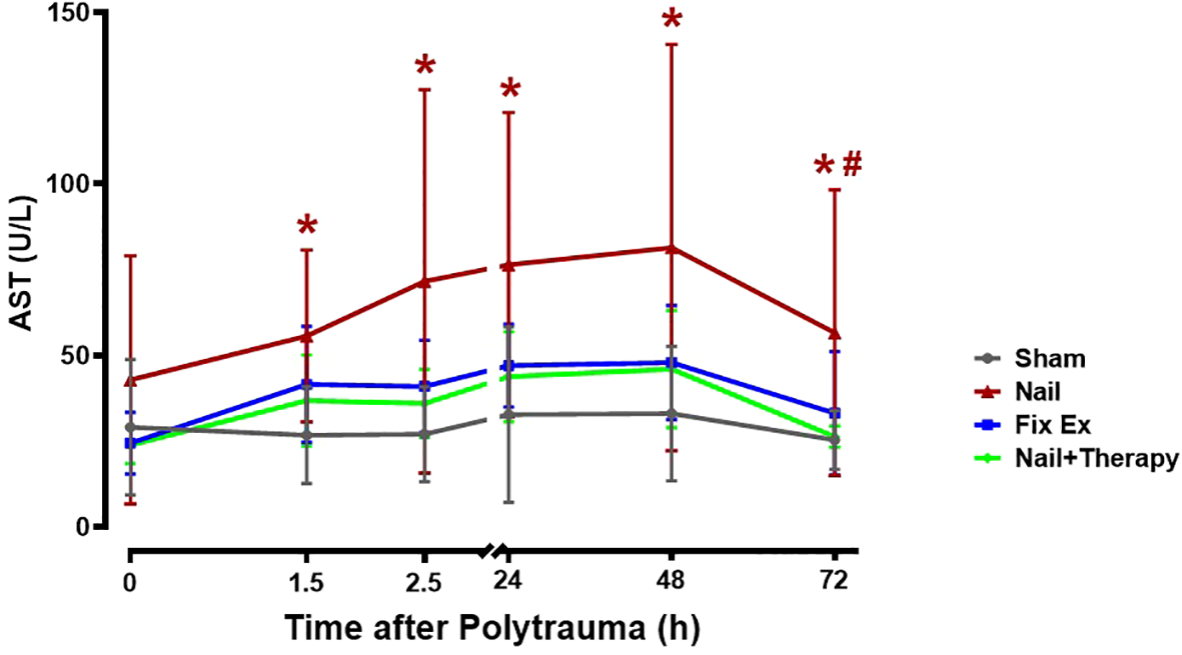
Effects of C5/CD14 blockade on enhanced serum aspartate transaminase (AST) concentrations after polytrauma. Blood samples were obtained from polytraumatized pigs at the indicated time-points. The Nail group displayed a higher overall serum AST concentration than the Nail+Therapy group (p<0.05), the latter group being similar to the Fix ex group without immunomodulation. By trend, AST levels in all groups increased until they peaked 48 h after trauma. AST levels at different time points for each group. Data are shown as the mean ± SD. Fix ex, external fixation; Nail, internal fixation with intramedullary nail; Nail+Therapy, intramedullary nail with combined C5/CD14 inhibitor treatment. Sham: n = 6, Fix ex: n = 8, Nail: n = 7, Nail+Therapy: n = 4. # denotes significant differences vs. Sham group. * denotes significant differences vs. 614 Nail+Therapy group.

Directly after the polytrauma and corresponding surgical intervention (0 h), except in the Nail group, there was no significant increase in serum AST levels (**Figure 2**). At 1.5 h after polytrauma, the AST level increased by trend in the Fix ex group and significantly in the Nail group compared to the Sham group (**Figure 2**; p<0.05), suggesting that the liver was significantly injured by 1.5 h after trauma, particularly in case of early total care (Nail). The changes were time dependent, peaking for all groups at 48 h after the trauma with a subsequent decrease (**Figure 2**). The AST serum concentrations in the Nail group decreased by immunomodulation at least to the AST levels found in the minimal invasive Fix ex group (**Figure 2**).

### 3.3 Immunomodulation reduces MPO positive cells in the post polytrauma liver

**Figure 3:**
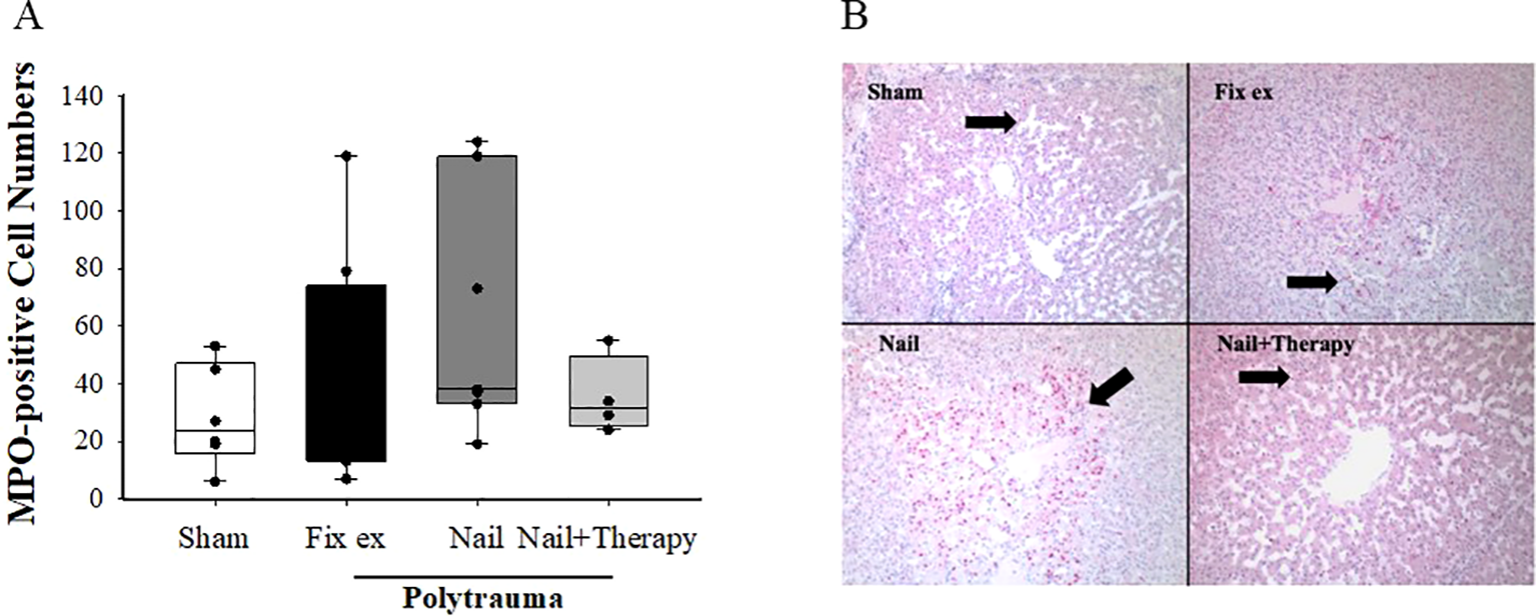
Amelioration of myeloperoxidase (MPO) staining of liver tissues by C5/CD14 inhibition after polytrauma. At 72 h after polytrauma or sham procedure, liver sections were stained for MPO and evaluated. Overall mean score of the number of MPO positive cells in each group **(A)**. ANOVA with Holm-Sidak posthoc testing was applied to compare the data between the groups. Data are displayed as means ± SD, and for the histological analysis as the median with the 25^th^/75^th^ percentile, denotes significant differences vs. Sham group, Representative histological sections at 100× magnification **(B)**. Black arrow: MPO positive cells in the hepatic lobules. Fix ex, external fixation; Nail, internal fixation with intramedullary nail; Nail+Therapy, intramedullary nail with combined C5/CD14 inhibitor treatment. Sham: n = 6, Fix ex: n = 8, Nail: n = 7, Nail+Therapy: n = 4.

After immunohistochemical staining, seven observation fields were randomly chosen for each liver tissue sample, and the MPO-positive cells were recorded using Image J to determine the mean number of such cells in each group (**Figure 3**). Few MPO positive cells were observed in liver tissues of sham pigs, whereas their numbers by trend in the liver tissues of polytraumatised pigs treated by either the Nail or Fix ex. By trend, a decrease in MPO staining with an overall reduced variance was found in livers from the Nail+Therapy versus the Nail only group (**Figure 3**). Addressing the anaphylatoxin C5a as a potent chemoattractant for inflammatory cells, the liver tissue concentrations of C5a were measured by ELISA, but did not significantly alter between the groups (**Supplementary Figure**).

### 3.4 Polytrauma increases hepatic expression and protein concentration of RANK, which is abolished by C5/CD14 inhibition

Subsequently, the gene expression and protein concentrations of RANK, RANKL, and OPG, as established key regulators of bone resorption and (re)modulation, were determined.

**Figure 4:**
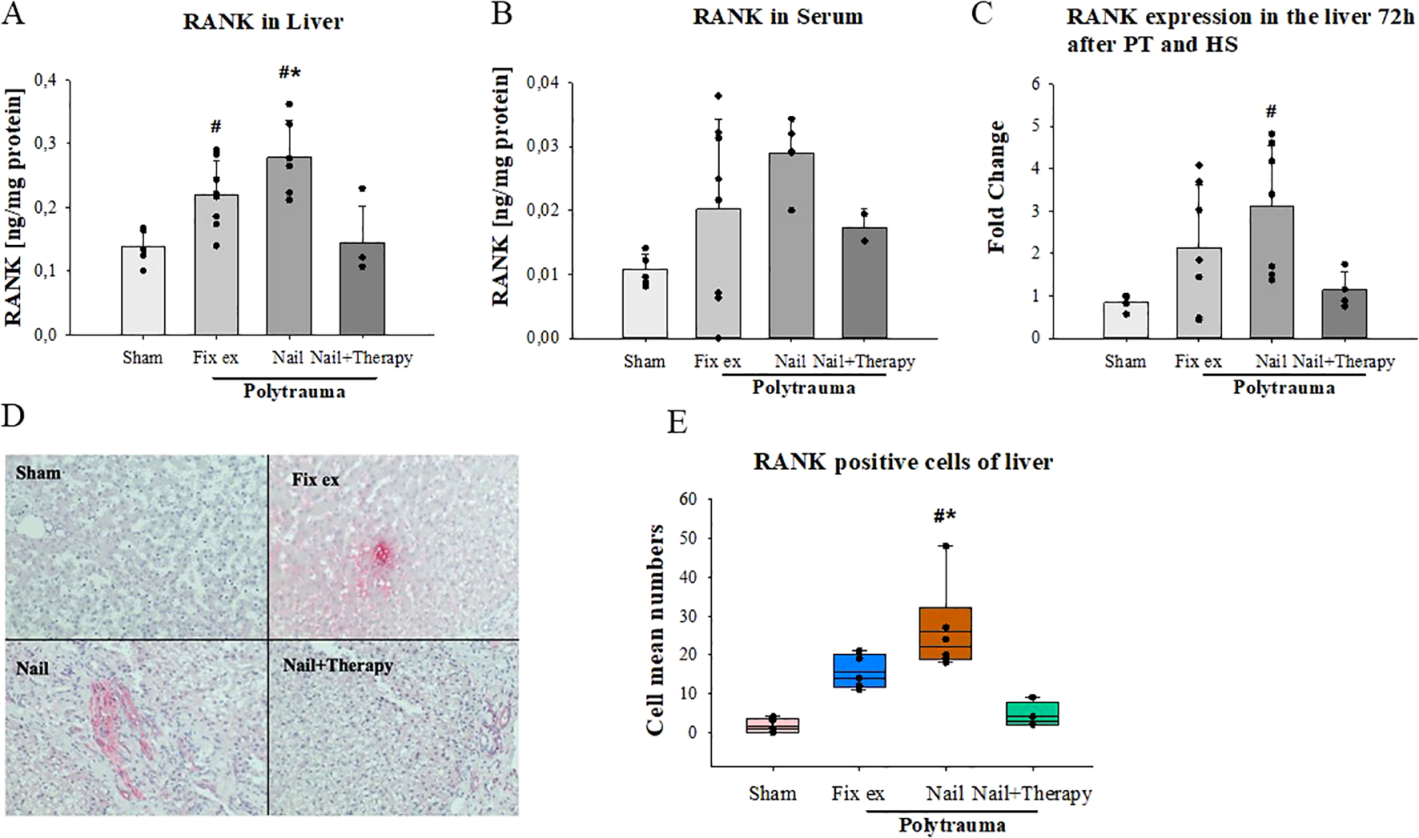
Detection of RANK in liver tissue and serum after polytrauma and reduction by systemic C5/CD14 blockade. RANK protein concentrations in liver tissue homogenates **(A)** and serum **(B)** 72 h after polytrauma (determined by ELISA). RANK RNA-expression levels in liver homogenates 72 h post trauma **(C)**. Representative histological liver staining images for RANK **(D)**. Statistical analysis of RANK positive cells in the liver histology **(E)**. Data satisfying a normal distribution were subjected to ANOVA, otherwise the rank sum test was applied. Data are shown as the mean ± SD; denotes significant differences vs. Sham group, * denotes significant differences vs Nail+Therapy group. Fix ex, external fixation; Nail, internal fixation with intramedullary nail; Nail+Therapy, intramedullary nail with combined C5/CD14 inhibitor treatment. Sham: n = 6, Fix ex: n = 8, Nail: n = 7, Nail+Therapy: n = 4.

On the protein level, slightly enhanced RANK concentrations were found in the liver (**Figure 4A**) and in serum (**Figure 4B**) post polytrauma, which, in the liver, were significantly higher in the Nail group than in the Nail+Therapy group. A similar pattern was found in the liver for the RANK RNA expression (**Figure 4C**). Immunohistochemical staining indicated some RANK protein staining in hepatocytes, particularly after polytrauma (**Figure 4D**). The number of RANK positive cells differed between the four groups, with the number of such cells in the Nail group being significantly higher than in the Sham and Nail+Therapy group (p<0.05) (**Figure 4E**).

### 3.5 Inhibition of C5/CD14 modulates liver and serum RANKL and OPG after polytrauma

**Figure 5:**
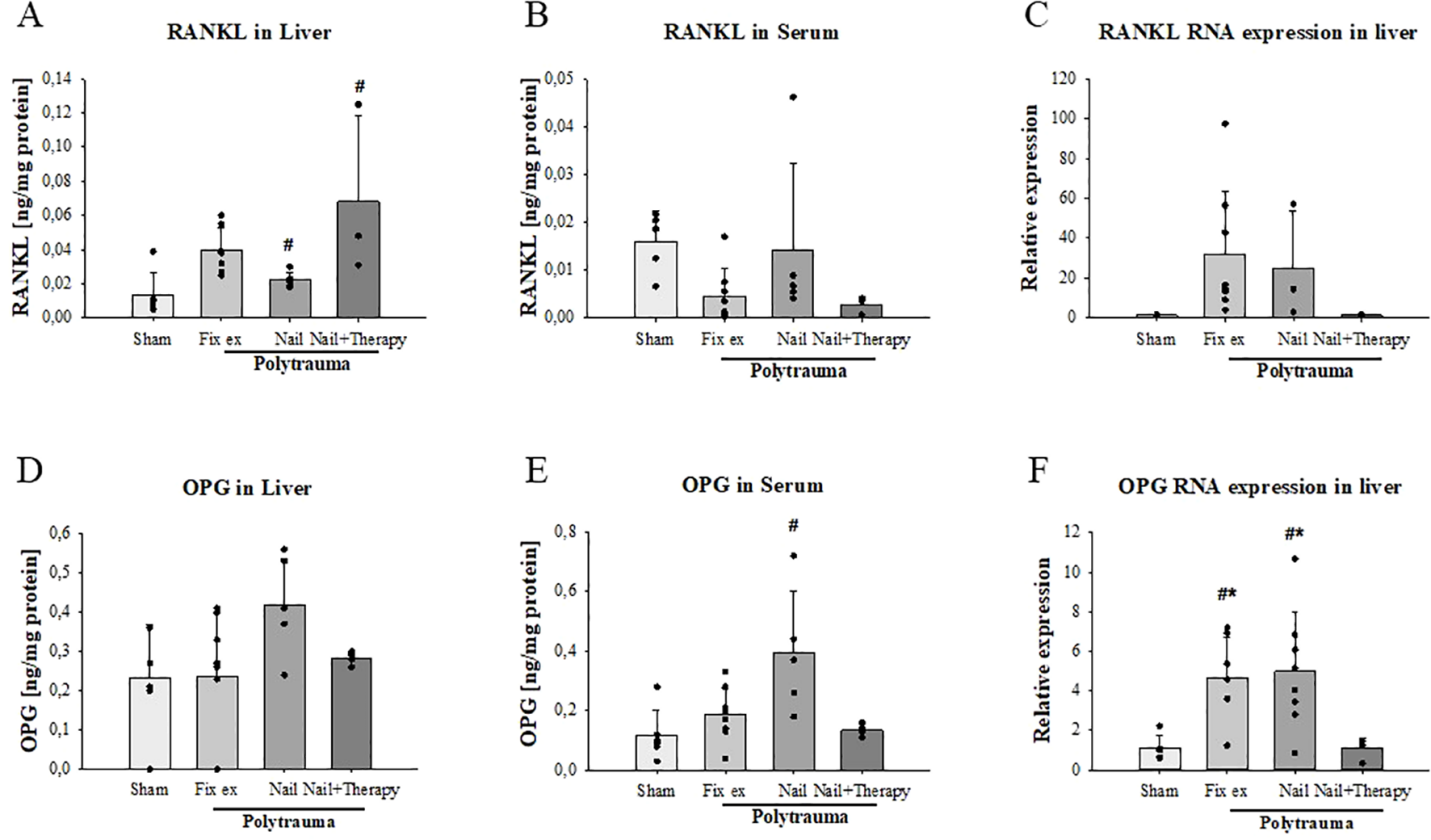
Reduction of polytrauma-induced changes in hepatic and systemic RANKL and OPG by inhibition of C5/CD14 in experimental polytrauma. RANKL and osteoprotegerin (OPG) serum protein concentration and hepatic RNA expression were determined 72 h after polytrauma in the indicated surgical treatment groups with and without immunomodulation of C5 and CD14. RANKL protein concentrations were determined by ELISA in liver tissue homogenates **(A)** and serum samples **(B)**. RANKL RNA expression levels in liver homogenates **(C)**. OPG protein concentrations in the liver **(D)** and serum **(E)** as well as OPG RNA expression in liver homogenates **(F)**. Data satisfying a normal distribution were subjected to ANOVA, otherwise the rank sum test was applied. Data are shown as the mean ± SD; denotes significant differences vs. Sham group, * denotes significant differences vs Nail+Therapy group. Fix ex, external fixation; Nail, internal fixation with intramedullary nail; Nail+Therapy, intramedullary nail with combined C5/CD14 inhibitor treatment. Sham: n = 6, Fix ex: n = 8, Nail: n = 7, Nail+Therapy: n = 4.

Regarding RANKL, liver protein concentrations were by trend slightly but insignificantly higher in the Fix ex group and slightly but significantly higher in the Nail group in comparison to the Sham group (p=0.014) (**Figure 5A**). The Nail+Therapy group exhibited slightly but significantly increased hepatic RANKL concentrations than in the Sham group (p=0.025). The RANKL serum concentrations were insignificantly altered, with some reduction in the Nail+Therapy group (**Figure 5B**). RANKL RNA expression was by trend, but insignificantly, enhanced post trauma in the Fix ex and Nail groups, which was abolished upon additional C5/CD14 inhibition (**Figure 5C**). These findings were associated with the limitation of a broad variance and small n-size in the therapy group.

Regarding OPG as a decoy receptor of RANKL, OPG protein was by trend higher in the liver (**Figure 5D**) and significantly higher in the serum after polytrauma and almost back to sham levels when comparing the Nail group in the absence versus the presence of the dual C5/CD14 inhibition (**Figure 5E**). With respect to the OPG RNA expression, the levels were approximately four times higher in the polytrauma groups without immunomodulation, but was abolished in the case of additional C5/CD14 blockade (**Figure 5F**).

## 4 Discussion

In the present study, a well-established, randomised, clinically relevant porcine polytrauma model with haemorrhagic shock was investigated (33). We focused on the liver-bone axis to investigate the effect of a dual immunomodulation.

The drug RA101295 (2-kDa peptide) given in the present study is a broad-spectrum C5 inhibitor. It is characterised by blocking C5 cleavage and the subsequent formation of anaphylatoxin C5a and terminal C5b–9, the latter found in two forms: sC5b–9 in the liquid phase and the MAC on the cell surface. C5a amplifies leukocyte activation and migration, induces basophil/mast cell degranulation, enhances vascular permeability, and therefore can induce all classical signs of inflammation (36). Blockade of the C5a-C5a receptor (C5aR)-interaction significantly improved IRI in the liver, characterised by the inhibition of platelet aggregation in the hepatic microcirculation and high mobility group box 1-release in the early stages of reperfusion. It also inhibited hepatocyte apoptosis by downregulating infiltrating macrophages and neutrophils, cytokine and chemokine release, and reactive oxygen species production (16). In a rodent study of distant organ damage after lower limb IRI, enhanced serum concentrations of lactate dehydrogenase (LDH), alanine transaminase (ALT) and AST and liver tissue tumor necrosis factor (TNF) were all reduced after application of a small peptide C5aR antagonist, indicating a key role of complement activation in the induction of remote liver damage (37). Mechanistically, C5a mediates leukocyte activation and migration during liver injury, ultimately leading to hepatocyte necrosis and apoptosis. In addition, the formed MAC can lyse target cells, promoting the release of further injury-associated molecular patterns, chemokines and other cytotoxins (16).

CD14 as a key molecule of TLRs has hardly been studied in polytrauma, as compared to complement. The TLRs are a major branch of innate immunity recognizing danger molecules, like the complement system. CD14 is a key player as co-receptors for several TLR molecules, in humans documented for TLR4 and TLR-2, and in mice for several others (21). Specific anti-CD14 mAbs to block porcine CD14 are scares. We found that mAb MIL-2 was promising in attenuating the cytokine response in *E. coli* sepsis (38). However, this antibody of the mouse subclass IgG2b showed some adverse effects due to the Fc part and we therefore genetically engineered a chimeric mouse-human mAb based on the human IgG2/4 Fc part (rMIL-2), avoiding complement activation and binding to Fc-receptors (31), which later have shown to be efficient in pig polymicrobial sepsis both with respect to morbidity and mortality (32). In a recent study, we demonstrated that a combined blockade of C5 and CD14 early in the posttraumatic course with an immune-monitoring-based real-time dose adjustment significantly reduced multiple organ damage (30). Focusing on the liver in the present study, as expected, we found enhanced liver damage scores including increased AST levels after PT surgically addressed by minimal invasive external fixation and even more by early total care with an intra-medullar femur nail. By contrast, a significant decrease of morphological and biochemical signs of liver damage and dysfunction was observed in this polytraumatized group after synchronic and combined C5/CD14 inhibition. Of note, in case of systemic AST concentrations, the immunomodulatory therapy was capable of reducing this liver injury marker in the early total care setting (Nail) to a level found in damage control surgery by the external fixateur (Fix ex), suggesting a promising therapeutic approach which could enable definitive treatment in combination with C5/CD14 inhibition.

Major trauma can cause compromised fracture repair. In a rat model of severe trauma, impaired fracture healing occurred in a surgical group with femoral osteotomy plus blunt thorax trauma compared to a group with femoral osteotomy alone (39). The acute systemic inflammatory response appears to alter the cellular composition and cytokine generation in the fracture haematoma, which substantially reduces bone formation and the mechanical competence of the fracture callus in the later stages of healing (40). On a cellular level, during the early inflammatory phase of fracture healing, C5aR was strongly expressed not only by immune cells, but also by osteoblasts, chondrocytes and osteoclasts in the intramembranous and chondrogenic zones of the fracture callus indicating a modulatory role of activated complement (41).

For fracture healing, the RANK-RANKL-OPG axis is important. Therefore, we investigated in the current study, to our knowledge, for the first time the liver expression of these mediators after PT and hemorrhagic shock (HS). In this context, we also addressed the effects of C5/CD14 inhibition on hepatic expression of these molecules. Our data revealed a consistent trend of enhanced RANK protein expression in the liver and serum 72 h post trauma. The RANKL/RANK interplay activates NF-kB in hepatocytes, leading to inflammatory cytokine production, Kupffer cell activation and increased fat storage (28). In a mouse model of liver IRI, serum RANKL concentrations were increased, peaking after 4 h, while OPG (as decoy receptor of RANKL) increased steadily over the observation period of 8 h after IRI. In the liver, RANK was constitutively expressed in hepatocytes and less in Kupffer cells. Of note, exogenous RANKL application revealed some protective liver effects after murine IRI (42). In our porcine model of polytrauma-induced liver injury, we found sustained protein generation of RANKL, RANK and OPG as late as 72 h post trauma. As a limitation of the study, we did not investigate the dynamics of the histological changes of the liver response. Even so, the C5/CD14 inhibition reduced the hepatic expression levels of RANK and OPG, effects that only can be speculated on to be somehow protective, because reduced OPG levels may lead to enhanced biological activity of RANKL in the liver. Activated T cells and other cells of the pro-inflammatory phenotype, including endothelial cells and lymphocytes are also major sources of RANKL (43, 44), which may contribute to the systemic response in addition to the postulated liver- bone communication. In a murine model of trauma with hemorrhagic shock reduced systemic RANKL levels in plasma at 24 and 72 hours post-trauma were measured, which aligns with our findings (45). However, it remains unclear why C5/CD14 blockade led to a reverse pattern concerning hepatic and systemic RANKL, and to what extent the polytrauma conditions or the various RANKL-generating cell types influence systemic RANKL levels. Further studies are needed to elucidate these mechanisms.

A liver-bone crosstalk was previously described in chronic liver diseases (46), for example, in hepatic osteodystrophy development (47). Despite its crucial function in bone remodelling, the role of the RANK-RANKL-OPG system (28) in the polytrauma pathophysiology remains unclear. In the present study, liver RANK of the PT Nail group was significantly higher than in the PT Nail+Therapy group. Furthermore, PCR results indicated higher OPG and RANKL gene expression in liver samples from the PT Nail group in comparison to the PT Nail+Therapy group.

Regarding the therapeutic approach, experimental studies have shown that inhibition of complement activation before the induction of liver injury results in hepatic protection, manifested by reduced inflammation and cell apoptosis (48–50). The liver is the major source of complement components. However, also osteoblasts can generate and activate complement proteins such as C3 and C5 (51–53). The complement cleavage products C3a and C5a have been shown to stimulate RANKL expression in osteoblasts, thereby increasing osteoclast formation (54). *In vivo*, increased osteoclast precursor cell recruitment to inflammatory sites may enhance the induction of osteoclast formation due to the chemotactic effects of the anaphylatoxins (55). Immunosuppression of C5a and C3a not only directly affects osteoclast formation, but also regulates osteoblast/osteoclast interactions via RANKL/OPG (54). However, it is noteworthy, that fracture repair also requires the involvement of the terminal complement complex (56). Through the current effective C5/CD14 inhibition, we reduced damaging effects of the inflammatory response on the liver, but at the same time, we also have inhibited C5b-9 generation and thereby potentially compromised bone and tissue regeneration and the clearance of damaged/infected cells (56, 57).

The innovation of the present study is the combined use of C5/CD14 inhibitors to alter inflammation and potentially key bone modulating factors in a clinically relevant long-term model of porcine polytrauma (observation period of 72 h). Through C5/CD14 double blockade, we found significant reduction of polytrauma-caused liver damage and inflammation, not only as assessed morphologically but also biochemically and on a transcription level. While the immunomodulatory approach revealed some protection on the posttraumatic liver response, it also significantly altered the hepatic and systemic RANK-RANKL-OPG axis.

## 5 Conclusion

In summary, we conclude:

- I Experimental polytrauma leads to liver injury and hepatic modulation of RANKL, RANK, and OPG;
- Seventy-two hours after trauma, the combined inhibition of C5/CD14 resulted in reduced polytrauma-induced liver injury and the hepatic generation of mediators, which can influence bone repair;
- The consequences of the liver-bone-axis on fracture healing requires further investigation.

## Data Availability

The original contributions presented in the study are included in the article/**Supplementary Material**. Further inquiries can be directed to the corresponding author.

## Supplementary material

Supplementary Table List of applied primers.

Supplementary Figure Concentration of the complement activation product C5a in liver tissue samples of the corresponding experimental groups: Sham: n = 6, Fix ex: n = 8, Nail: n = 7, Nail+Therapy: n = 4.
