# Supplementary figures and images for: Modulation of the hepatic RANK-RANKL-OPG axis by combined C5 and CD14 inhibition in a long-term polytrauma model
Source: Front Immunol. 2024 Nov 21;15:1434274. doi: 10.3389/fimmu.2024.1434274 (PMC11617561; doi:10.3389/fimmu.2024.1434274)

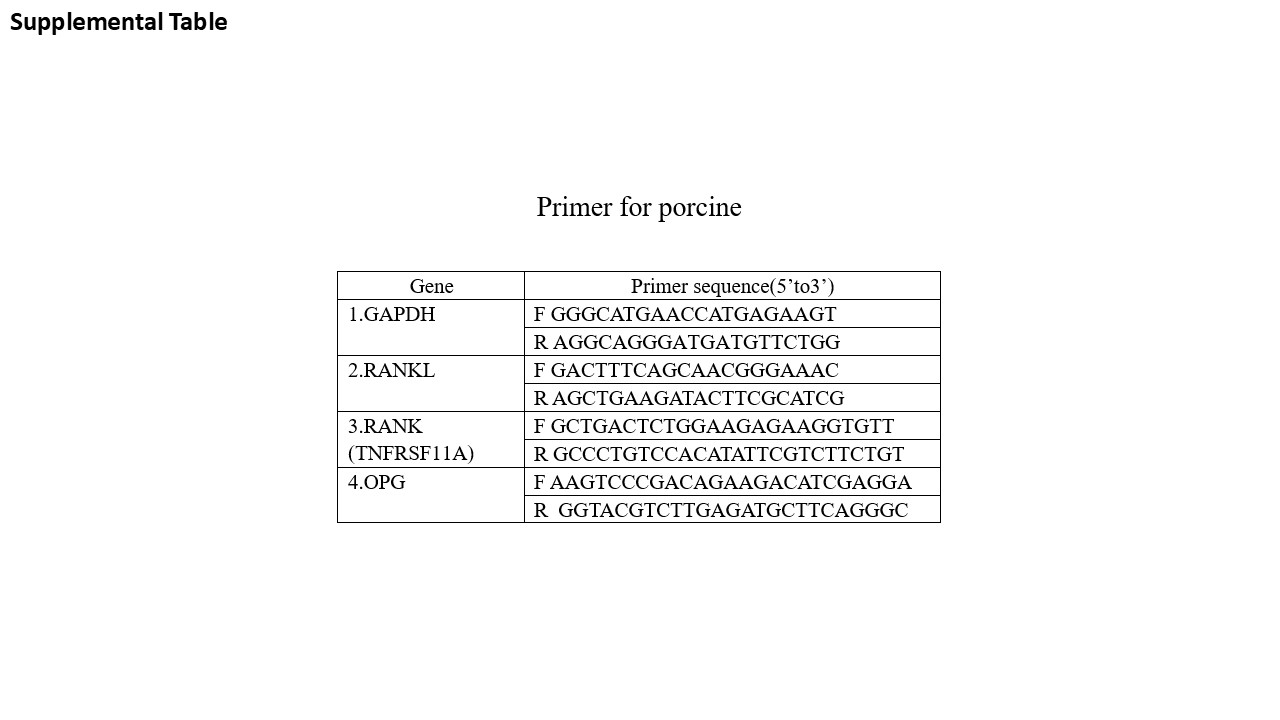

Supplement: Supplementary Table — List of applied primers. [file Image2.jpeg]

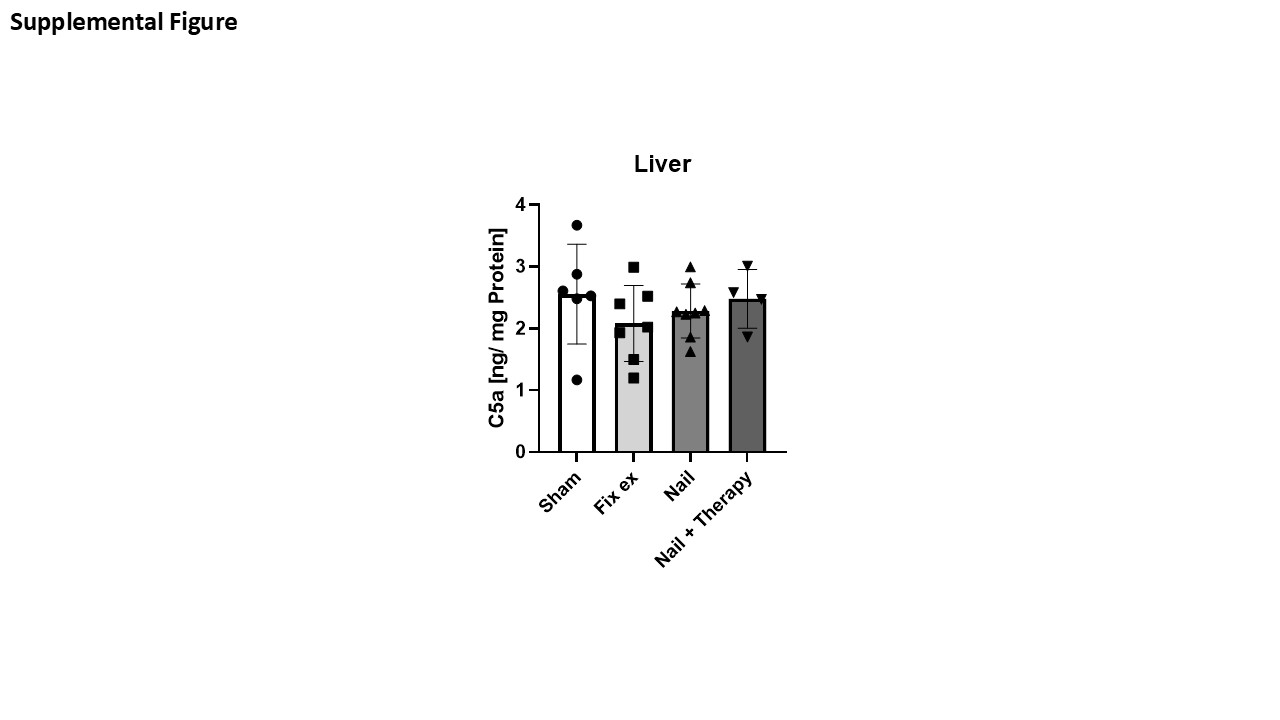

Supplement: Supplementary Figure — Concentration of the complement activation product C5a in liver tissue samples of the corresponding experimental groups: Sham: n = 6, Fix ex: n = 8, Nail: n = 7, Nail+Therapy: n = 4. [file Image1.jpeg]
